# Overexpressing the Myrosinase Gene *TGG1* Enhances Stomatal Defense Against *Pseudomonas syringae* and Delays Flowering in Arabidopsis

**DOI:** 10.3389/fpls.2019.01230

**Published:** 2019-10-04

**Authors:** Kaixin Zhang, Hongzhu Su, Jianxin Zhou, Wenjie Liang, Desheng Liu, Jing Li

**Affiliations:** College of Life Sciences, Northeast Agricultural University, Harbin, China

**Keywords:** *Arabidopsis*, flowering, myrosinase, *Pst* DC3000, stomatal defense

## Abstract

Myrosinase enzymes and their substrate glucosinolates provide a specific defensive mechanism against biotic invaders in the Brassicaceae family. In these plants, myrosinase hydrolyzes glucosinolates into diverse products, which can have direct antibiotic activity or function as signaling molecules that initiate a variety of defense reactions. A myrosinase, β-thioglucoside glucohydrolase 1 (TGG1) was previously found to be strikingly abundant in guard cells, and it is required for the abscisic acid (ABA) response of stomata. However, it remains unknown which particular physiological processes actually involve stomatal activity as modulated by TGG1. In this experimental study, a homologous *TGG1* gene from broccoli (*Brassica oleracea* var. *italica*), *BoTGG1*, was overexpressed in *Arabidopsis*. The transgenic plants showed enhanced resistance against the bacterial pathogen *Pseudomonas syringae* pv. *tomato* (*Pst*) DC3000 *via* improved stomatal defense. Upon *Pst* DC3000 infection, overexpressing *BoTGG1* accelerated stomatal closure and inhibited the reopening of stomata. Compared with the wild type, *35S::BoTGG1* was more sensitive to ABA- and salicylic acid (SA)-induced stomatal closure but was less sensitive to indole-3-acetic acid (IAA)-inhibited stomatal closure, thus indicating these hormone signaling pathways were possibly involved in stomatal defense regulated by TGG1. Furthermore, overexpression of *BoTGG1* delayed flowering by promoting the expression of *FLOWERING LOCUS C* (*FLC*), which encodes a MADS-box transcription factor known as floral repressor. Taken together, our study’s results suggest glucosinolate metabolism mediated by TGG1 plays a role in plant stomatal defense against *P. syringae* and also modulates flowering time by affecting the FLC pathway.

## Introduction

Glucosinolates are major secondary metabolites found in the Brassicales order, including the model plant *Arabidopsis* and many vegetables (Agerbirk and Olsen, 2012). Myrosinase (β-thioglucoside glucohydrolase, TGG) hydrolyzes glucosinolates by cleaving the thioglucosidic bond to release hydrolysis products that are toxic to insects and pathogens (Tierens et al., 2001; Hopkins et al., 2009; Calmes et al., 2015). Glucosinolates and myrosinase are normally harbored in separate compartments within plants (Andreasson et al., 2001; Koroleva et al., 2010). But they come into contact with each other upon tissue disruption from chewing by insects or damaged by pathogens rapidly releasing large amounts of toxic hydrolysis products, typically isothiocyanates and nitriles and their derivatives (Halkier and Gershenzon, 2006).

Myrosinase activity has been detected in all glucosinolate-containing plants (Husebye et al., 2002). So far, six *TGG* genes encoding classical myrosinases have been found in the model plant *Arabidopsis* (Xu et al., 2004). The *TGG1* and *TGG2* genes are expressed aboveground, where the myrosinases encoded by each are considered to mainly break down aliphatic glucosinolates (Xue et al., 1995; Barth and Jander, 2006); *TGG3* and *TGG6* are pseudogenes having multiple frame-shift mutations in their coding regions, being specifically expressed in the plant’s anthers (Husebye et al., 2002; Zhang et al., 2002); finally, *TGG4* and *TGG5* are specifically expressed in the roots and related to auxin synthesis and root-growth regulation (Fu et al., 2016). In addition to these six classical TGG myrosinases, two other β-glucosidases, PEN2 and PYK10, were identified as atypical myrosinases that primarily hydrolyze indole glucosinolates (Bednarek et al., 2009; Nakano et al., 2017).

Numerous studies have demonstrated that the glucosinolate–myrosinase defense system supports broad-spectrum immunity in *Arabidopsis*. Besides generating direct antimicrobial activity through toxic hydrolysis products, glucosinolate degradation can also form signaling molecules to initiate conserved defense responses. Clay et al. (2009) found that degradation of tryptophan-derived indole glucosinolate mediated by atypical myrosinase PEN2 is required for callose deposition in pathogen-associated molecular pattern (PAMP)-triggered immunity (PTI). Likewise, TGG1 and TGG2 are both presumed to be involved in conserved immune responses against pathogens. For example, the hydrolysis of methionine-derived aliphatic glucosinolates mediated by TGG1 and TGG2 is required for programmed cell death (PCD) upon inoculation with the bacterial pathogen *Pseudomonas syringae* pv. *tomato* (*Pst*) DC3000 and the downy mildew *Hyaloperonospora arabidopsidis* (Andersson et al., 2015). Interestingly, the indole glucosinolate degradation, as catalyzed by TGG1, attenuates mycotoxin fumonisin B1 (FB1)-induced PCD (Zhao et al., 2015); this suggests that glucosinolates’ metabolism responds to different kinds of pathogens through various molecular mechanisms.

Apart from influencing PCD, TGG1 and TGG2 also contribute to stoma activity in *Arabidopsis*. As the most abundant stomatal protein, TGG1 comprises 40% to 50% of the total proteins found in guard cells; accordingly, the *tgg1* mutant has impaired wound-induced stomatal closure and is less sensitive to abscisic acid (ABA)-inhibited opening of its stomata (Zhao et al., 2008), while the *tgg1/tgg2* double mutant is defective in both ABA and methyl jasmonate (MeJA)-induced stomatal closure (Islam et al., 2009). Consistent with those findings, some glucosinolate-derived isothiocyanates were found capable of inducing stomatal closure (Hossain et al., 2013; Sobahan et al., 2015). Taken together, these studies indicate that myrosinase activity and corresponding hydrolysis products play a critical role in the regulation of stomatal closure. Yet it remains elusive which particular physiological processes TGG-modulated stomatal activity is involved in.

Glucosinolate metabolism contributes to not only defense against biotic stress but also plant development. Several reports have suggested that glucosinolates and their metabolites are involved in the modulation of flowering time in *Arabidopsis* (Naur et al., 2003; Kerwin et al., 2011; Jensen et al., 2015; Kong et al., 2015; Xu et al., 2018). Both *AOP2* and *AOP3* are genes in the aliphatic glucosinolate biosynthesis pathway, encoding two 2-oxoglutarate-dependent dioxygenases that modify glucosinolate side chains (Kliebenstein et al., 2001). These two paralogous genes possess the ability to affect glucosinolate accumulation and flowering time; however, they differ in their ability to modulate flowering time, so the two genes may affect the flowering phenology *via* separate mechanisms (Jensen et al., 2015). In particular, *AOP2* can alter the circadian clock pathway, but whether this contributes to regulated flowering time is questionable (Kerwin et al., 2011). Although cross talk does occur between glucosinolate metabolism and flowering control, many unknown processes await further exploration.

In this study, transgenic *Arabidopsis* plants overexpressing a homologous *TGG1* gene from broccoli (designated here as *BoTGG1*) were investigated, from which several interesting phenotypes were observed. We also performed the same analysis in plants overexpressing endogenous *AtTGG1*; similar phenotypes were observed but none as significant as *35S::BoTGG1*. To more clearly expound on the function of myrosinase TGG1, only data concerning *35S::BoTGG1* are presented here.

We found that *35S::BoTGG1* plants were more resistant against the bacterial pathogen *Pst* DC3000. Given that TGG1 participates in stomatal activity, we hypothesized that this enhanced pathogen resistance present in 35S::BoTGG1 arose from an altered stomatal defense. Stomata are natural openings on the surface of leaves, which not only enable the gas exchange but also facilitate the entry of bacteria. Hence, stomatal closure is considered among the conservative immune mechanisms plants employ against bacterial pathogens (Melotto et al., 2006; Zeng et al., 2010; Sawinski et al., 2013). When bacteria attack, the plant first recognizes PAMP and responds by closing its stomata. To circumvent this immune response, bacteria may release a specific virulence factor to effectively cause stomata to reopen (Melotto et al., 2006). In our study, we discovered that overexpression of *BoTGG1* accelerated stomatal closure and inhibited stomatal reopening upon infection of *Pst* DC3000. Furthermore, *35S::BoTGG1* was more sensitive to ABA- and salicylic acid (SA)-induced stomatal closures while less sensitive to indole-3-acetic acid (IAA)-inhibited stomatal closure, indicating that TGG1-affected stomatal defense likely operates *via* the signaling pathway of these hormones. In addition to enhanced pathogenic resistance, *35S::BoTGG1* displayed another phenotype, that of delayed flowering, which led to significant increases in the biomass of both the aerial part and root system.

Considering that high biomass and pathogen resistance are important plant breeding goals, our study will prove useful for breeding economically valuable cruciferous vegetables with both traits by modifying their glucosinolate metabolism.

## Materials and Methods

### Plant Material and Growth Conditions

Seeds of broccoli (*Brassica oleracea* var. *italica*) cultivar ‘Qingxiu’ were purchased from the JiaHe Seeds Company (Beijing, China) and used for *BoTGG1* cloning. Seeds of *Arabidopsis* ecotype Columbia (Col-0) were obtained from the Arabidopsis Biological Resource Center and used for genetic transformation. All plants were grown under a 16-h photoperiod, with a photosynthetic photon flux density of 100 μmol·m^−2^·s^−1^, at a 23°C temperature and 60% relative humidity.

### Molecular Cloning and Plant Transformation

Total RNA was extracted from 3-day-old broccoli seedlings using the EASYPure Plant RNA Kit (TransGen, Beijing, China). The cDNA was synthesized from total RNA with the PrimeScript RT-PCR Kit (Takara, Shiga, Japan). The coding sequence (CDS) of the *BoTGG1* gene was amplified by using the primers BoTGG1-F and BoTGG1-R (primer sequences are listed in **Table S1**). To construct the expression vector *35S::BoTGG1*, the obtained PCR product was cloned into the expression vector pCAMBIA330035Su according to a previously described method (Nour-Eldin et al., 2006).

The constructed expression vector was passed through the *Agrobacterium tumefaciens* strain *LBA4404* and transferred into *Arabidopsis via* inflorescence infection (Clough and Bent, 1998). To select the transformants, seeds were planted on the 1/2 Murashige and Skoog (MS) agar medium containing 50 mg L^−1^ of kanamycin. Two independent homozygous transgenic lines were used in the subsequent analyses.

### Glucosinolate Extraction and Analysis

The *35S::BoTGG1* and wild-type plants were grown simultaneously for 4 weeks. For each plant, 100–150 mg of rosette leaves was harvested for the glucosinolate extraction according to a previously described method (Hansen et al., 2007). Glucosinolates were extracted with methanol, and the desulfoglucosinolates were obtained by filtration through a DEAE Sephadex column followed by sulfatase treatment. High-performance liquid chromatography (HPLC) analysis was carried out as previously described (Grosser and van Dam, 2017). Glucosinolates were identified as desulfoglucosinolates, with sinigrin used as the external standard.

### Myrosinase Determination

Rosette leaves of 4-week-old wild-type plants and two independent transgenic lines of *35S::BoTGG1* were harvested for their myrosinase activity assay. These fresh leaves (150 mg) were frozen in liquid nitrogen and quickly ground into powder. This ground sample was solubilized in 1-ml extraction buffer (pH 7.2) containing 10 mM of K-phosphate, 1 mM of EDTA, 3 mM of dithiothreitol (DTT), and 5% glycerol and then vortexed and centrifuged at 12,000 × *g* for 15 min at 4°C. The supernatant was collected to measure myrosinase activity.

Myrosinase activity was quantified by calculating the rate of hydrolysis of sinigrin. For this, the reaction buffer consisted of 33.3 mM of K-phosphate (pH 6.5) and 0.2 mM of sinigrin; the reaction was initiated by adding 100-μl extracted enzyme into 2.9 ml of reaction buffer. The decline in absorbance at 227 nm and 37°C spanning a 5-min period was plotted. Myrosinase activity was evaluated as the amount of sinigrin degraded by the enzyme from 1 g of fresh leaf per minute.

### Bacterial Growth Assay

The virulent pathogen *Pst* DC3000 was used, with bacteria cultured in King’s B medium at 28°C. For the sprayed infection, 4-week-old plants were sprayed with a bacterial suspension of *Pst* DC3000 [10^8^ colony-forming units (CFU) ml^−1^] in 10 mM of MgCl_2_ containing 0.04% Silwet L-77. For infection by injection, leaves were syringe-infiltrated with a bacterial suspension of *Pst* DC3000 (10^6^ CFU ml^−1^) in 10 mM of MgCl_2_. Those plants sprayed or injected with only 10 mM of MgCl_2_ served as the corresponding control. The inoculated plants were kept at high humidity for 3 days. The amount of bacterial growth in the infected leaves was determined as described (Katagiri et al., 2002).

### Measurement of Stomatal Aperture

Four-week-old plants were used for the stomatal aperture bioassay. Peels of rosette leaves were first floated in an opening buffer containing 5 mM of KCl, 50 mM of CaCl_2_, and 10 mM of MES-Tris (pH = 6.15) under light for 3 h to induce the maximum opening of the stomata. For the bacterial infection, leaf peels were transferred to a water suspension of *Pst* DC3000 (10^8^ CFU ml^−1^), while those moved to water alone served as the control. Stomatal aperture was observed every 15 min during a 3-h period. For the ABA, SA, and MeJA treatments, leaf peels were respectively transferred to the opening buffer with 10 μM of ABA, 500 μM of SA, and 10 μM of MeJA for 2 h. For the IAA treatment, two groups of leaf peels were transferred to a new opening buffer solution, with or without IAA addition, and then placed under darkness for 2 h. Leaf peels likewise transferred to the opening buffer without hormone additions served as the control. Width and length of each stoma were measured using ImageJ software, and stomatal apertures were expressed by their width-to-length ratio.

### Quantitative Real-Time PCR Analysis

To detect transcript levels of genes involved in the glucosinolate biosynthesis pathway, rosette leaves from 4-week-old plants were harvested. To detect transcription levels of genes involved in stomatal defense, detached leaves from 4-week-old plants were incubated with a water suspension of *Pst* DC3000 (10^8^ CFU ml^−1^). Leaves incubated with water served as the corresponding control. The incubated leaves were collected every 15 min during a 1-h period. To detect transcript levels of genes involved in flowering, rosette leaves from 18-day-old plants were harvested. For all sets of leaves, their total RNA was isolated using the TRIzol reagent (Invitrogen, Carlsbad, CA). The first strand of cDNA was synthesized using the PrimeScript RT Reagent Kit (Takara, Shiga, Japan), and quantitative real-time PCR (qRT-PCR) analyses were performed using the Trans Start Top Green qPCR SuperMix (TransGen, Beijing, China) on an ABI 7500 sequence detection system. The detected genes and the primers used in qPCR are listed in **Table S1**. The *ACTIN2* gene in *Arabidopsis* served as the internal reference gene. The gene expression level was calculated according to the 2^−ΔΔCt^ method.

### Biomass Determination

For biomass determination, mature plants that had completely developed and grown after they produced their terminal flowers were used. The aerial tissues and seeds were harvested respectively and dried at 70°C for 2 days. The dry weight of each plant was measured, and the relative weight of wild type and *35S::BoTGG1* was calculated.

### Detection of Drought Resistance

Wild-type and *35S::BoTGG1* plants were grown under a long-day condition (16-h photoperiod) for 4 weeks. Detached rosette leaves from *35S::BoTGG1* and the wild type were placed on a piece of weighing paper, and the fresh weight of these leaves was measured every 20 min during a 3-h period. Water loss was defined as the percentage of initial weight reduced at each time point.

The same amount of soil (by weight) was placed into each pot, after which all pots were soaked on a tray to ensure equivalent soil and water conditions among them. Seeds of *35S::BoTGG1* and the wild type were planted and allowed to grow for 4 weeks, with water added to the tray twice a week. To emulate drought, water was withheld from plants for 2 weeks, and then all the plants were rewatered for 2 days. Plant growth under each water condition was photographed and observed.

## Results

### Cloning of *BoTGG1* From Broccoli

According to our previous transcriptome analysis in broccoli (Gao et al., 2014), the CDS of a myrosinase gene was amplified from the broccoli (*B. oleracea* var. *italica*) cultivar ‘Qingxiu’, by using the unigene *TGG* as the reference sequence. The CDS of the obtained gene was 1,647 nucleotides long, encoding a protein of 548 amino acids; this revealed 98.4% nucleotide and 99.2% amino acid identity when compared to the predicted myrosinase gene in broccoli (*B. oleracea* var. *oleracea*). Compared to its homologous *TGG* genes in *Arabidopsis*, the obtained gene was most similar to *TGG1*, with which it shared 71.5% nucleotide and 79.5% amino acid identity. We therefore designated the obtained gene as *BoTGG1* (GenBank accession no. MG252789).

### Overexpression of *BoTGG1* Increased Myrosinase Activity and Decreased Aliphatic Glucosinolate Content in *Arabidopsis*

To confirm whether *BoTGG1* possesses myrosinase activity as its homolog in *Arabidopsis*, *BoTGG1* was overexpressed in *Arabidopsis*, and this overexpression of *BoTGG1* was confirmed by RT-PCR (**Figure S1**).

**Figure 1:**
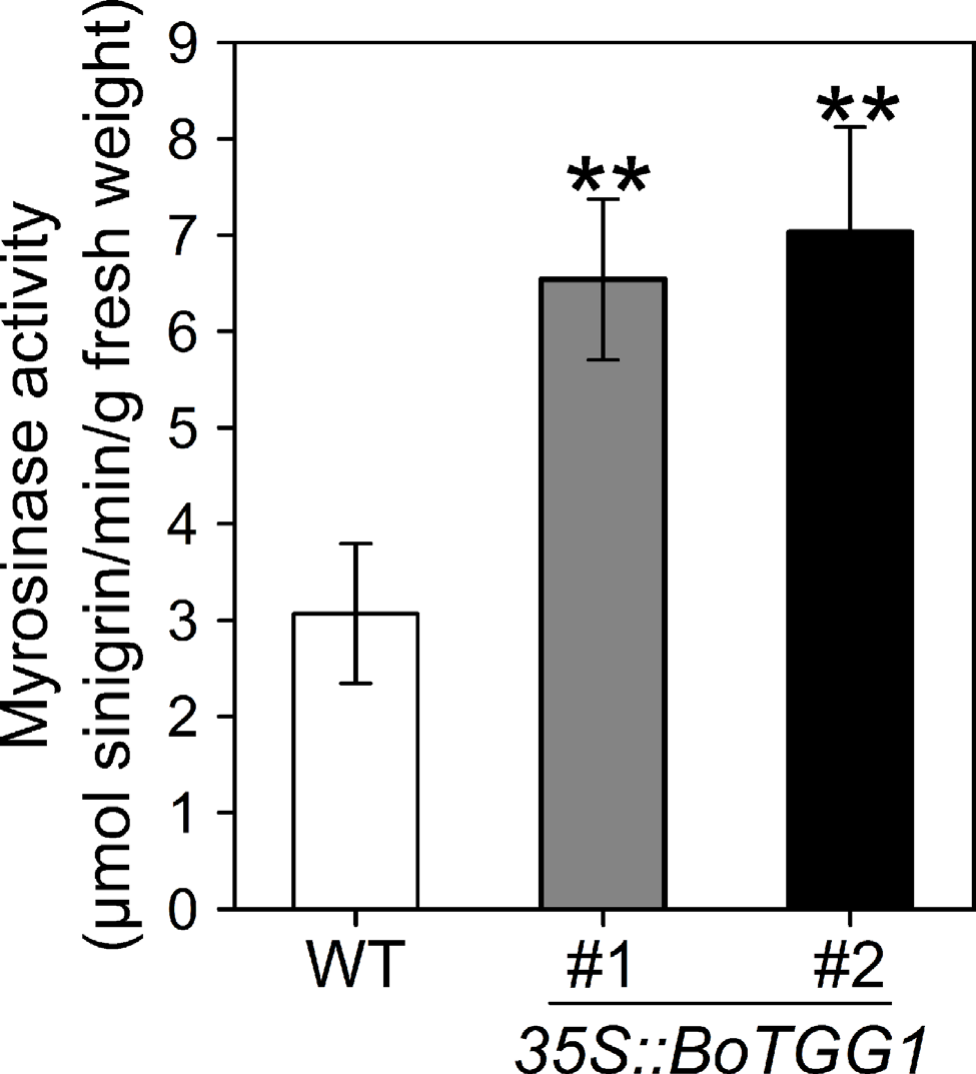
Myrosinase activity of wild type (WT) and *35S*::*BoTGG1*. Rosette leaves of 4-week-old WT and two independent transgenic lines of *35S*::*BoTGG1* were harvested for myrosinase activity assay. Means (± SE) from three independent biological replicates and three technical repeats are shown. **, significantly different (Student’s *t*-test; *P* < 0.01) from the WT.

Rosette leaves from 4-week-old *35S::BoTGG1* and wild-type plants were harvested, and the myrosinase activity assay was performed using sinigrin (2-propenyl glucosinolate) as the substrate. Compared with wild-type plants, myrosinase activity in *35S::BoTGG1* was significantly increased (**Figure 1**), demonstrating that *BoTGG1* is functional *in vitro*.

**Figure 2:**
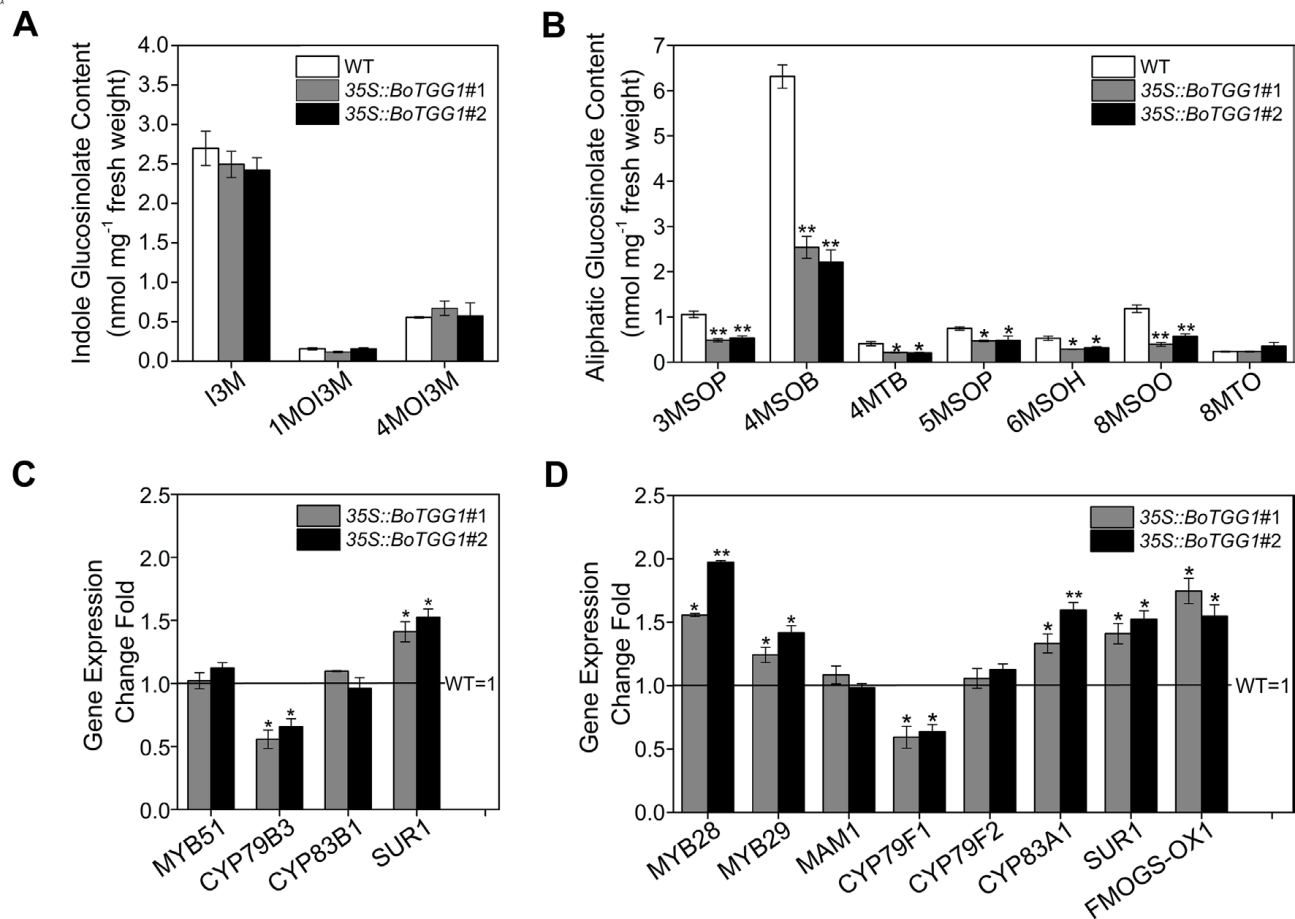
Glucosinolate profiles and expression of genes in the glucosinolate biosynthetic pathway. **(A)** Content of indole glucosinolates. I3M, indol-3-ylmethyl-glucosinolate; 1MOI3M, 1-methoxyindol-3-ylmethyl-glucosinolate; 4MOI3M, 4-methoxyindol-3-ylmethyl-glucosinolate. **(B)** Content of aliphatic glucosinolates. 3MSOP, 3-methylsulfinylpropyl-glucosinolate; 4MSOB, 4-methylsulfinylbutyl-glucosinolate; 4MTB, 4-methylthiobutyl-glucosinolate; 5MSOP, 5-methylsulfinylpentyl-glucosinolate; 6MSOH, 6-methylsulfinylhexyl-glucosinolate; 8MSOO, 8-methylsulfinyloctyl-glucosinolate; 8MTO, 8-methylthiooctyl-glucosinolate. **(C)** Relative expression level of genes in the indolic glucosinolate biosynthetic pathway. **(D)** Relative expression level of genes in the aliphatic glucosinolate biosynthetic pathway. Rosette leaves of 4-week-old wild type (WT) and two independent transgenic lines were harvested for analysis of their glucosinolate contents and quantitative real-time PCR. Relative expression values are given in comparison with the WT (WT = 1). Means (± SE) from three independent biological replicates and three technical repeats are shown. * and **, significantly different (Student’s *t*-test; *0.01 < *P* < 0.05, **P < 0.01) from the WT.

To further detect the effects of overexpressing *BoTGG1* on the glucosinolate profile *in vivo*, the glucosinolate content of rosette leaves from wild-type and *35S::BoTGG1* plants was measured. In *35S::BoTGG1*, the content of indole glucosinolates did not show any significant difference, but aliphatic glucosinolates were significantly reduced to approximately half that in the wild type (**Figures 2A, B**). This decrease of aliphatic glucosinolates in *35S::BoTGG1* was possibly due to increased myrosinase activity, but it could also have arisen from decreased biosynthesis of these compounds. To determine which, the expression levels of key genes involved in glucosinolate biosynthesis were assessed. Comparing with the wild type, in *35S::BoTGG1* plants, expression levels of the indole glucosinolate biosynthetic genes *MYB51* and *CYP83B1* were apparently not altered, while *CYP79B3* and *SUR1* were slightly changed (**Figure 2C**). For the genes related to aliphatic glucosinolate biosynthesis, the respective expressions of *MYB28*, *MYB29*, *CYP83A1*, *SUR1*, and *FMO_GS-OX1_* were all slightly increased, whereas *MAM1* and *CYP79F2* were not changed and *CYP79F1* slightly decreased (**Figure 2D**). Nevertheless, since the altered expression of each gene was less than twofold, we may infer that these genes’ expression during both indole and aliphatic glucosinolate biosynthesis in *35S::BoTGG1* remained unchanged compared with the wild type. This suggested the lowered content of aliphatic glucosinolates in *35S::BoTGG1* ought to be due to enhanced myrosinase activity and corresponding degradation processes. In sum, these results indicated that under normal growing conditions, the overexpressed *BoTGG1* primarily broke down aliphatic glucosinolates in intact tissues of *Arabidopsis*.

### Overexpression of *BoTGG1* Enhanced Resistance to *Pst* DC3000

**Figure 3:**
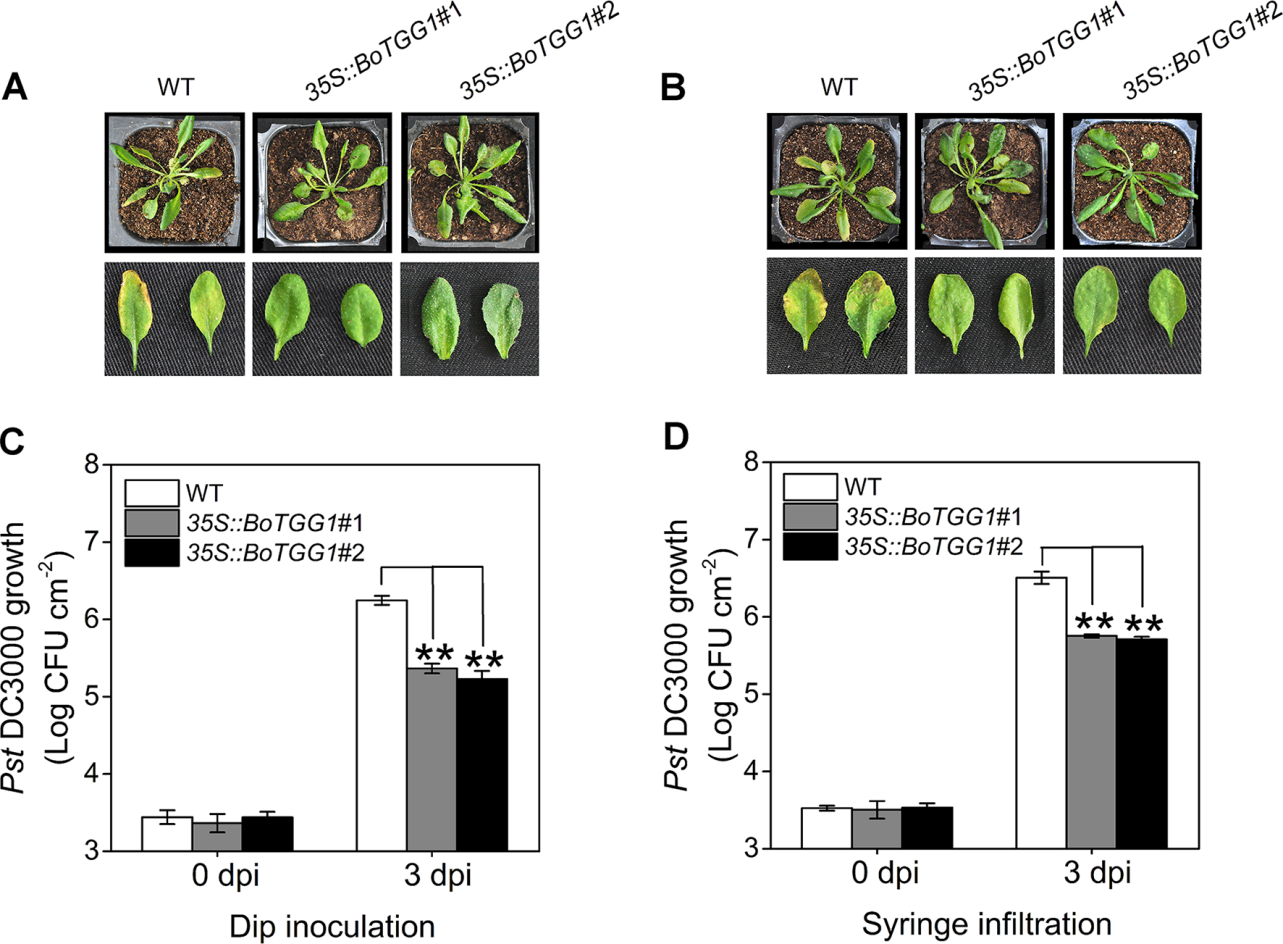
Enhanced resistance of *35S*::*BoTGG1* infected with *Pseudomonas syringae* pv. *tomato* DC3000 (*Pst* DC3000). The wild type (WT) and *35S::BoTGG1* were dip-inoculated with a suspension of *Pst* DC3000 (10^8^ CFU ml^−1^) and syringe-infiltrated with a suspension of *Pst* DC3000 (10^6^ CFU ml^−1^), respectively. **(A)** Disease symptoms of plants infected with *Pst* DC3000 by dip inoculation. **(B)** Disease symptoms of plants infected with *Pst* DC3000 by syringe infiltration. The pictures were taken at 3 days after inoculation. **(C)** Bacterial growth on leaves infected with *Pst* DC3000 by dip inoculation. **(D)** Bacterial growth on leaves infected with *Pst* DC3000 by syringe infiltration. Means (± SE) from three independent biological replicates and three technical repeats are shown. **, significantly different (Student’s *t*-test; *P* < 0.01) from the WT. dpi, days post inoculation.

To determine whether overexpression of *BoTGG1* could affect pathogenic resistance in *Arabidopsis*, virulent *Pst* DC3000 was used as a representative pathogen, and dip inoculation and syringe infiltration assays were performed. Leaves of *35S::BoTGG1* and wild-type plants were infected with *Pst* DC3000. Three days after infection, for both dip inoculation and syringe infiltration, wild-type plants displayed clear chlorotic symptoms. In contrast, *35S::BoTGG1* plants showed no significant signs of infection (**Figures 3A, B**). The growth of bacteria in leaves of the wild type was approximately 10-fold higher than that of *35S::BoTGG1* in the dip assay and likewise sixfold higher in the syringe assay (**Figures 3C, D**). These results suggested that overexpression of *BoTGG1* enhanced resistance to *Pst* DC3000 in *Arabidopsis*. The difference in bacterium growth between the wild type and *35S::BoTGG1* was larger when inoculated on the surface than when inoculated directly into the apoplast; hence, it may be more difficult for bacteria to enter through the epidermis in *35S::BoTGG1*.

### Overexpression of *BoTGG1* Accelerated Stomatal Closure and Inhibited Stomatal Reopening Upon Infection of *Pst* DC3000

**Figure 4:**
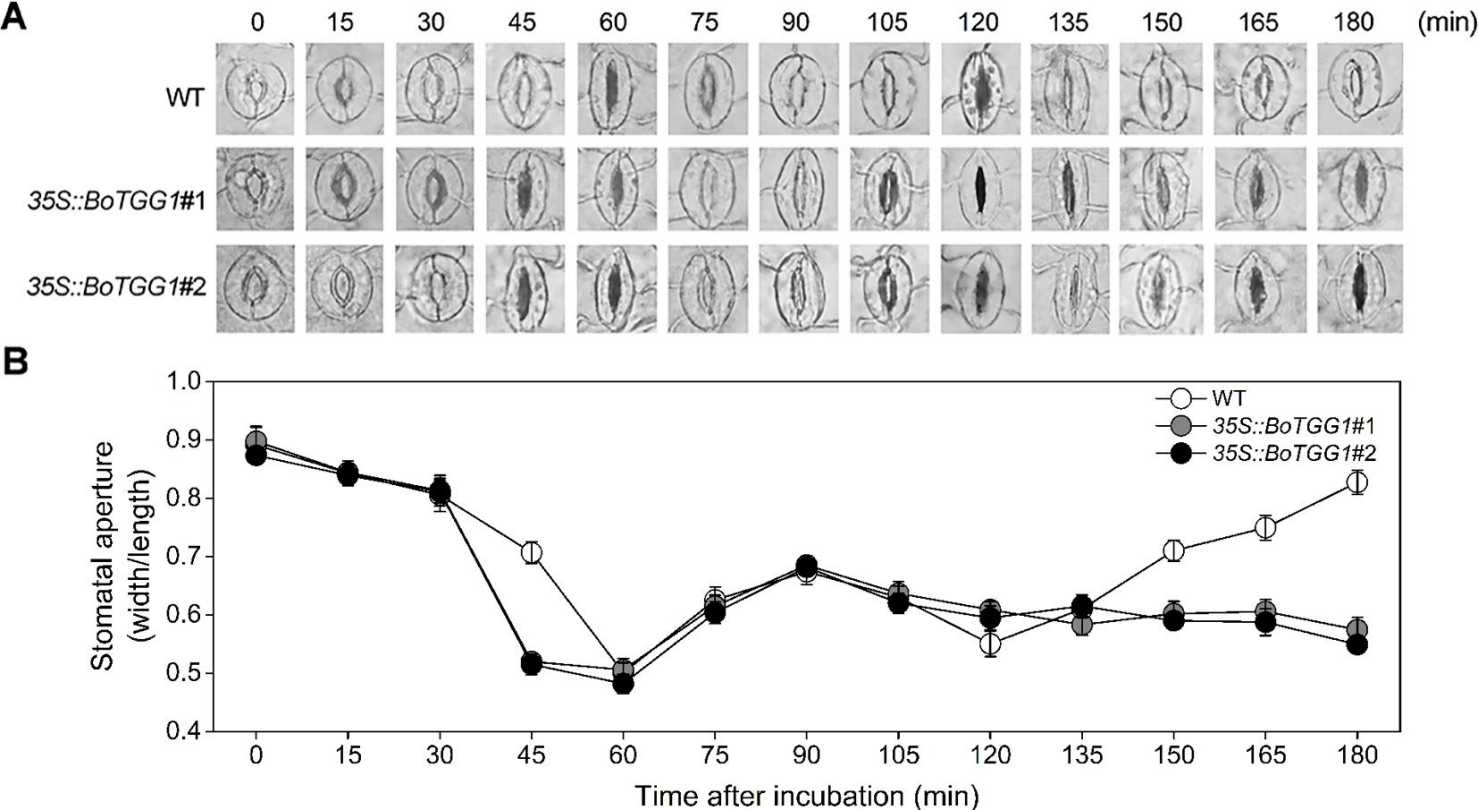
Stomatal movement in wild type (WT) and *35S*::*BoTGG1* upon infection of *Pseudomonas syringae* pv. *tomato* DC3000 (*Pst* DC3000). Leaf peels of WT and *35S::BoTGG1* plants were incubated with a suspension of *Pst* DC3000 (10^8^ CFU ml^−1^) for 3 h, and their stomatal apertures were observed and measured every 15 min. **(A)** The images of representative stomata at each time point during the infection. **(B)** Stomatal apertures at each time point during the infection. Means (± SE) from three independent experiments, each with 100 stomata, are shown.

Previous work discovered that *Arabidopsis* responds to the PAMPs of bacteria by closing its stomata ∼1 h after incubation with *Pst* DC3000, but this stomatal closure was transient, as bacteria subsequently produced a polyketide toxin, coronatine, which induced stomatal reopening ∼3 h after incubation to let them enter the host plant (Melotto et al., 2006). It is of great interest to know whether overexpression of *BoTGG1* can affect the closing and reopening of stomata during the arms race between plants and pathogenic bacteria. Therefore, we observed the dynamic state of stomata during the first 3 h of *Pst* DC3000 incubation. Water incubation was performed as control, and no stomatal movement was observed (**Figure S2**). In the wild type, 90% of stomata had completely closed by ∼60 min post inoculation, while in *35S:: BoTGG1* this extent of closure occurred earlier, at ∼45 min post inoculation. Within 60–120 min after the inoculations, half of the stomata had reopened, but not to their maximum apertures, and they were subsequently closed again at 120 min post inoculation in both wild-type and transgenic plants. No significant differences between the two genotypes were observed within this period. During the last 60 min, however, 90% of the stomata had reopened gradually in the wild type while most of the stomata remained closed in *35S::BoTGG1* (**Figures 4A, B**). These results suggested that overexpression of *BoTGG*1 accelerated *Pst* DC3000-induced closing of the stomata and inhibited their later reopening.

### *35S::BoTGG1* Was More Sensitive in ABA- and SA-Induced Stomatal Closure and Less Sensitive in IAA-Inhibited Stomatal Closure

**Figure 5:**
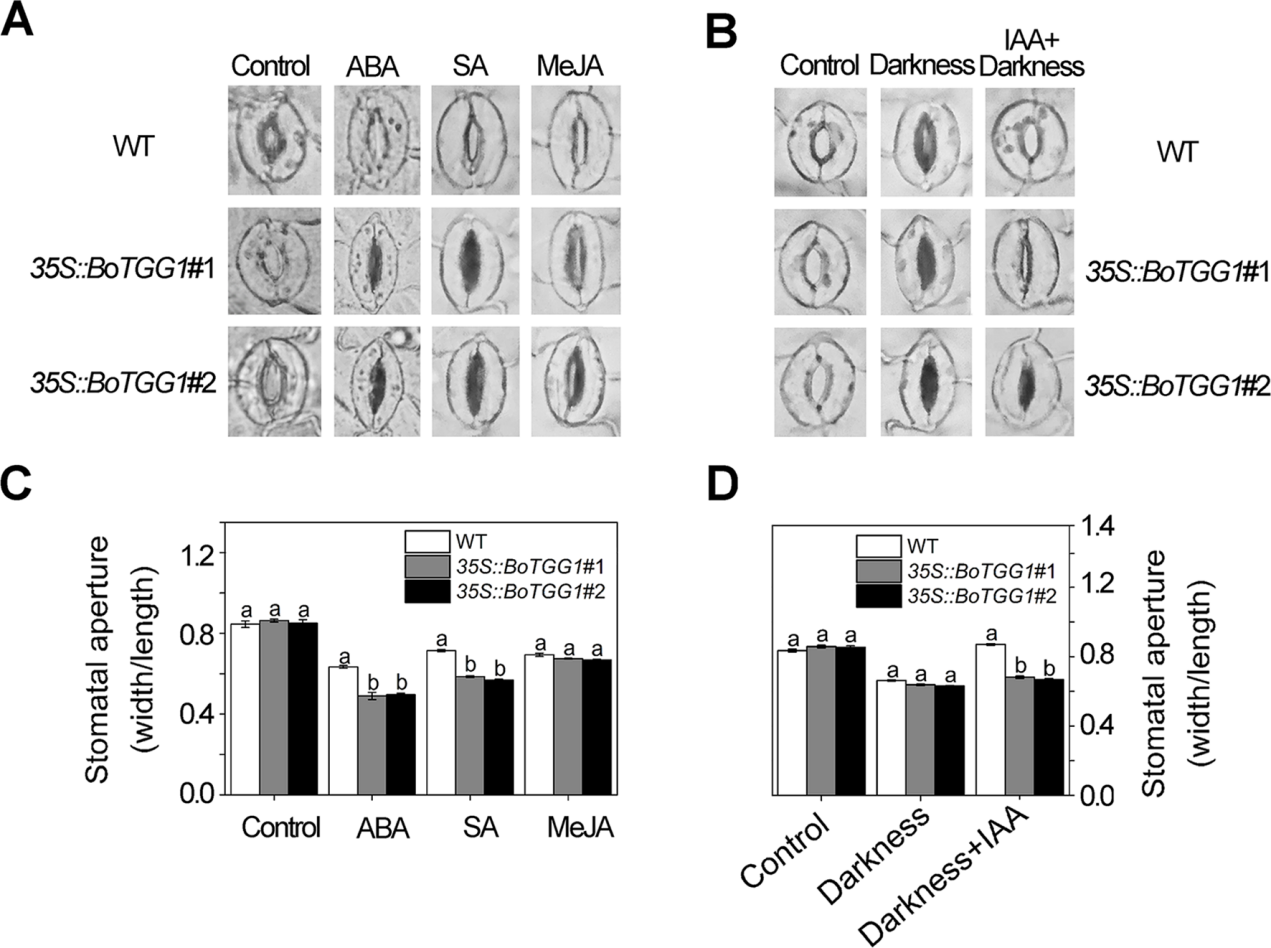
Stomatal responses to different phytohormones in wild type (WT) and *35S::BoTGG1*. **(A)** Images of representative stomata treated with abscisic acid (ABA), salicylic acid (SA), and methyl jasmonate (MeJA) hormones. Leaf peels of WT and *35S::BoTGG1* were respectively incubated with a stomatal opening buffer with 10 μM of ABA, 500 μM of SA, or 10 μM of MeJA under lighted conditions for 2 h. Leaf peels transferred to the opening buffer were used as the control. **(B)** Images of representative stomata treated with indole-3-acetic acid (IAA). Two groups of leaf peels (with maximum opening of stomata) were transferred to new opening buffer with or without IAA addition and left under darkness for 2 h. Leaf peels transferred to the same opening buffer lacking hormone addition were used as the control. **(C)** Stomatal apertures under treatment with ABA, SA, and MeJA. **(D)** Stomatal apertures under treatment with IAA. For stomatal apertures, means (± SE) from three independent experiments, each with 100 stomata, are shown. Different letters indicate significant differences (ANOVA test; *P* < 0.05) between the WT and *35S::BoTGG1* under the same treatment.

As shown in **Figures 5A, C**, ABA and SA effectively induced stomatal closure in both the wild type and *35S::BoTGG1*. Compared with wild-type plants, the stomatal aperture was significantly smaller in *35S::BoTGG1*, indicating the latter plant was more sensitive to ABA- and SA-induced closing of the stomata. Much like ABA and SA, JA was able to induce stomatal closure in both the wild type and 35S::*BoTGG1*, but no significant difference was observed between the two genotypes, suggesting that overexpression of *BoTGG1* did not affect stomatal closure induced by this hormone. IAA is another hormone that can positively regulate stomatal opening and is strongly induced by coronatine upon infection by pathogenic bacteria (Lohse and Hedrich, 1992; Kunkel and Harper, 2018). As **Figures 5B, D** show, in wild-type plants, stomata were largely closed under darkness in the absence of IAA but stayed open in its presence. In contrast, in *35S::BoTGG1*, the darkness led to stomatal closure irrespective of IAA being present or not. This result suggested that *35S::BoTGG1* was less sensitive to IAA-inhibited stomatal closure.

**Figure 6:**
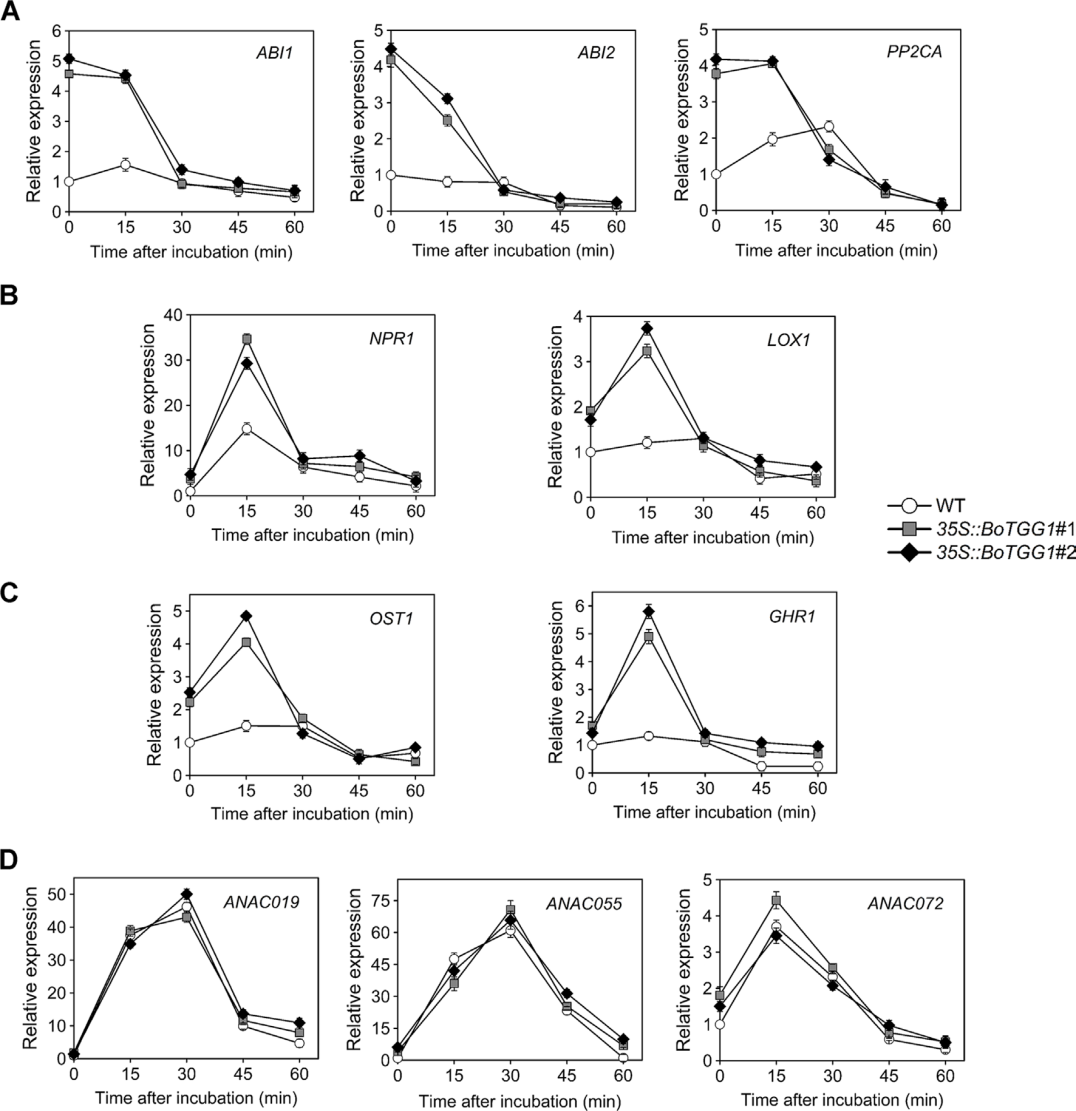
Relative expression of several stomatal defense-related genes in response to *Pseudomonas syringae* pv. *tomato* DC3000 (*Pst* DC3000) in the wild type (WT) and *35S*::*BoTGG1*. **(A)** Relative expression of genes in ABA-mediated stomatal closure pathway. **(B)** Relative expression of genes in SA-mediated stomatal closure pathway. **(C)** Relative expression of *OST1* and *GHR1*. **(D)** Relative expression of genes inducing stomatal reopening. Detached leaves after dip inoculation with a suspension of *Pst* DC3000 (10^8^ CFU ml^−1^) at the given time points were used for quantitative real-time PCR analysis. Transcript levels in the WT at 0 min was set to 1. Means (± SE) from three independent biological replicates and three technical repeats are shown.

ABA-mediated stomatal closure is involved in bacterium-triggered stomatal defense (Melotto et al., 2017). To determine whether ABA-mediated stomatal closure is more sensitive in *35S::BoTGG1* in response to *P. syringae*, we analyzed the expression levels of several pathogen defense-related genes in the ABA-mediated stomatal closure. Since bacterium-triggered stomatal defense is a fast response (<1 h) (Melotto et al., 2017), the gene expression levels were detected at five time points within an hour upon *Pst* DC3000 infection. ABI1, ABI2, and PP2CA are negative regulators of ABA signaling and play essential roles in pathogen resistance (Park et al., 2009; Rodrigues et al., 2013). The expression levels of the three genes were significantly higher in *35S::BoTGG1* than in the wild type before *Pst* DC3000 infection (**Figure 6A**). During the first hour of *Pst* DC3000 infection, the expression levels of *ABI1*, *ABI2*, and *PP2CA* in *35S::BoTGG1* decreased, while the expression levels of the three genes in the wild type did not change significantly. The decreased expression of these negative regulator genes indicated the activating of the ABA signaling pathway, and this might contribute to the faster stomatal closure in *35S::BoTGG1*.

The SA signaling pathway is also required for stomatal defense (Khokon et al., 2011). NPR1 is a major activator of SA-mediated responses and is essential for stomatal defense (Schellenberg et al., 2010). *LOX1*, a gene expressed in guard cells, encodes lipoxygenase, the catalytic product of which triggers SA-mediated stomatal closure (Montillet et al., 2013). As shown in **Figure 6B**, the expression of both *NPR1* and *LOX1* showed a rapid and transient increase during the first 30 min of *Pst* DC3000 infection. The expression level of the two genes in *35S::BoTGG1* increased much more than that in wild-type plants, which suggested SA-triggered stomatal response might be more sensitive in *35S::BoTGG1*.

*SLAC1* encodes a guard cell-expressed anion channel, which is a major contributor of stomatal closure. OST1 and GHR1 are two kinases that activate SLAC1 in parallel during ABA-induced stomatal closure, and GHR1 is also involved in SA-mediated stomatal closure (Hua et al., 2012; Acharya et al., 2013). Similar to what has been observed in *LOX1* and *NPR1*, a rapid and transient increase in the expression of *OST1* and *GHR1* was observed in *35S::BoTGG1* during the early response to *Pst* DC3000, while the expression increases of the two genes in wild type were not significant (**Figure 6C**). These results further suggested that in response to *Pst* DC3000, stomatal closure mediated by ABA and SA might be more sensitive in *35S::BoTGG1*.

ANAC019, ANAC055 and ANAC072 are three homologous NAC family transcription factors that are required for coronatine-induced stomatal reopening through JA signaling pathway (Zheng et al., 2012). As shown in **Figure 6D**, the expression of these three genes showed a significant and transient increase, but no significant difference were observed between *35S::BoTGG1* and the wild type. So ANAC019, ANAC055 and ANAC072 might not (or at least not at transcription level) contribute to the insensitive stomatal reopening in *35S::BoTGG1*.

Stoma’s movement is the most critical factor regulating water transpiration in plants, and is closely related to their drought resistance. Compared with the wild type, *35S::BoTGG1* exhibited no difference in the water loss of its detached rosette leaves and showed comparable growth under drought stress (i.e., withholding water for 2 weeks; **Figures S3A, B**). This result suggested that overexpressing *BoTGG1* enhanced stomatal closure in response to pathogen infection but not to drought stress, and thus it did not change the drought resistance of plants.

### Overexpression of *BoTGG1* Delayed Flowering Time

**Figure 7:**
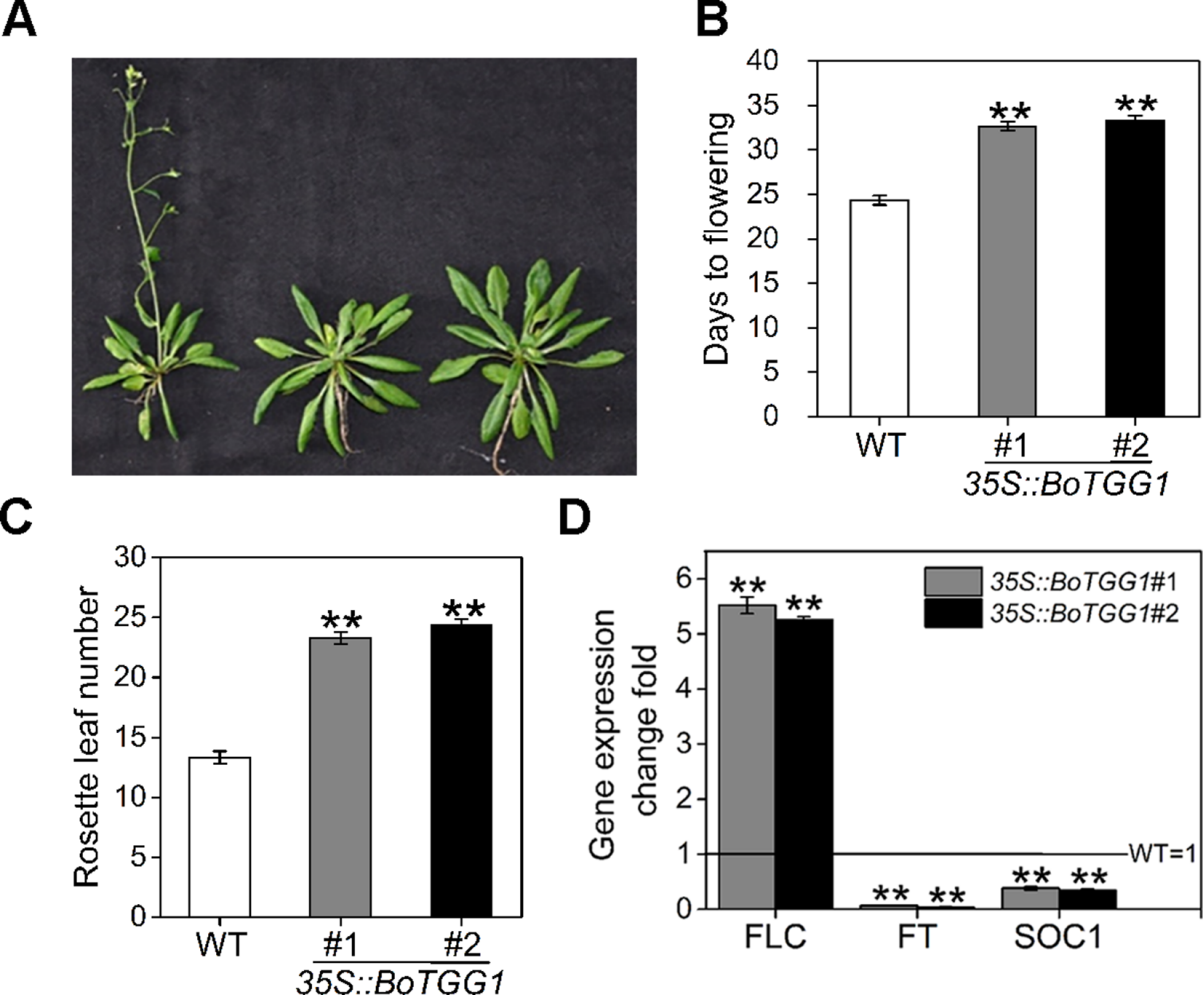
Delayed flowering in *35S::BoTGG1* plants. **(A)** Flowering phenotype in *35S::BoTGG1*. **(B)** Average flowering time (days after germination). **(C)** The average number of rosette leaves at time of flowering. **(D)** Relative expression levels of flowering-related genes in the wild type (WT) and *35S::BoTGG1*. Rosette leaves from 18-day-old plants grown under a 16-h photoperiod were harvested for quantitative real-time PCR analysis. Change fold of gene expression in comparison with the WT is shown. Means (± SE) from three independent biological replicates and three technical repeats are shown. **, significantly different (Student’s *t*-test; **P < 0.01) from the WT.

The mechanism by which glucosinolates modulate flowering time and whether myrosinase is involved in the modulation remains unclear. We found that overexpressing *BoTGG1* in *Arabidopsis* significantly delayed flowering under a long-day condition **(Figures 7A–C**). Compared with the wild type, *35S::BoTGG1* plants flowered about 8-9 days later. Recently, by using two *Aethionema arabicum* accessions with distinct glucosinolate profile, Mohammadin et al. (2017) identiﬁed a single major quantitative trait locus controlling total glucosinolate content; they suggested that *FLC* is a potential major regulator of glucosinolate content, and that a plant’s defense and its vegetative-to-reproductive stage transition can affect each other. To determine whether the delayed flowering in *35S::BoTGG1* was indeed related to a *FLC*-mediated flowering pathway, transcription levels of *FLC*, *FT*, and *SOC1* genes in rosette leaves of wild-type and *35S::BoTGG1* plants were detected. As **Figure 7D** shows, in *35S::BoTGG1* the expression of *FLC* significantly exceeded the wild type whereas that of *FT* and *SOC1* were significantly lower than in wild type. This indicated that an *FLC*-dependent flowering pathway might have contributed to the delayed flowering that characterized *35S::BoTGG1*.

**Figure 8:**
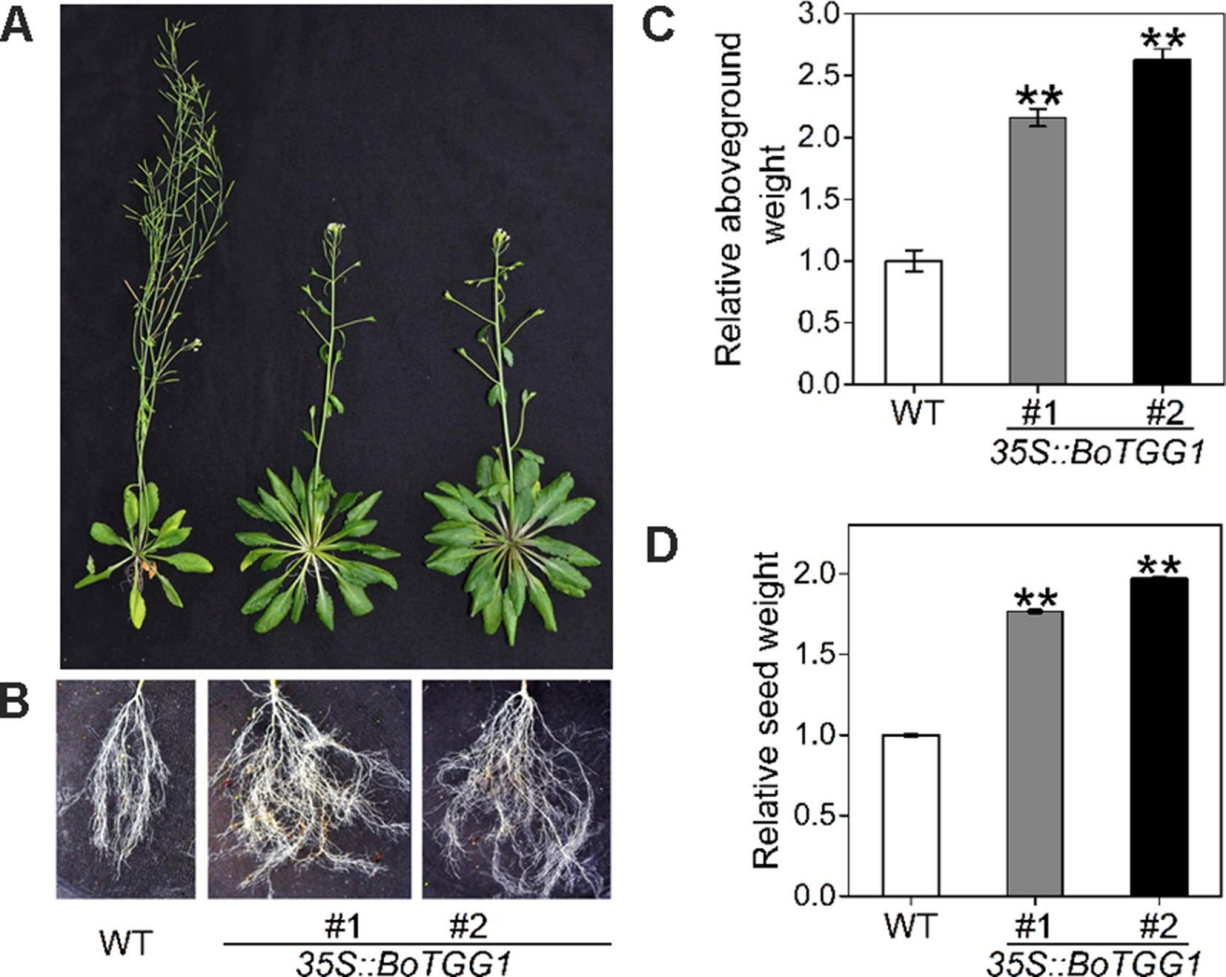
Biomass of wild type (WT) and *35S::BoTGG1*. **(A)** Aboveground parts of WT and *35S::BoTGG1*. **(B)** Roots of WT and *35S::BoTGG1*. **(C)** Relative aboveground dry weight of WT and *35S::BoTGG1*. **(D)** Relative seed dry weight of WT and *35S::BoTGG1*. Means (± SE) from measurements of more than five plants per genotype are shown. Relative weight of WT and *35S::BoTGG1* were measured after the plants produced their terminal flowers; for WT, these are 43-day-old plants, and for *35S::BoTGG1*, these are 71-day-old plants. **, significantly different (Student’s *t*-test; *P* < 0.01) from the WT.

Due to this delayed flowering, vegetative growth in *35S::BoTGG1* was prolonged by 9 days and its total growth phase prolonged ca. 28 days. Consequently, these transgenic plants appeared significantly larger in both their aerial and underground parts in the later stage of life cycle (**Figures 8A, B**). After terminal flower production, the final dry weight of aerial tissue and seeds of *35S::BoTGG1* respectively reached 2.4- and 1.9- fold that of wild-type counterparts (**Figures 8C, D**). However, an increased biomass in *35S::BoTGG1* before flowering could not be detected, indicating the improved biomass found before was due to a prolonged growth phase caused by delayed flowering.

## Discussion

Being the most common natural openings in plants, stomata are the first barriers bacterial pathogens must overcome to successfully colonize their host. Not surprisingly, plants have evolved ways to thwart such pathogen attack by closing their stomata rapidly upon detecting an infection in progress. To circumvent this plant immune response, bacteria have evolved a reciprocal strategy to effectively cause stomata to reopen, so they could penetrate the host (Melotto et al., 2006).

In this study, we found that enhanced myrosinase activity promoted plant resistance against a pathogenic bacterium (*Pst* DC3000) by accelerating the closure and inhibiting the reopening of stomata upon infection. Both ABA and SA play positive roles in stomatal closure and are thought to function in PAMP-induced stomatal closure (Melotto et al., 2017). In our study, *BoTGG1*-overexpressing plants were more sensitive to ABA-and SA-induced stomatal closure, thus indicating overexpression of *BoTGG1* promoted bacteria-induced stomatal closure possibly *via* the ABA and SA signaling pathways. The quantitative real time PCR analysis of the key genes in ABA- and SA- mediated stomatal closure pathways supported our speculation. In *Arabidopsis*, *TGG1* and *TGG2* are required in ABA- and MeJA-induced stomatal closure (Islam et al., 2009). We found that MeJA induced stomata to close in both wild-type and *35S::BoTGG1* plants, but no difference was detected between the two genotypes; this suggests altered TGG1 activity did not affect MeJA-mediated stomatal closure pathway as tested under our experimental conditions. Although MeJA-induced stomatal closure has been detected in some studies (Suhita et al., 2004; Munemasa et al., 2007; Arnaud et al., 2012; Hua et al., 2012; Yan et al., 2015), this could not always be verified by other research groups (Montillet et al., 2013; Savchenko et al., 2014). In fact, the role of JA-Ile as an inhibitor of stomatal closure is more strongly supported (Staswick and Tiryaki, 2004; Sellam et al., 2007; Panchal et al., 2016). In response to plant stomatal defenses, bacterium-produced coronatine mimics JA-Ile to induce stomatal reopening. This inconsistency is explained in that MeJA-induced stomatal closure might depend on reaching an endogenous ABA threshold (Hossain et al., 2011).

Generally, IAA plays a positive role in regulating the opening of stomata (Lohse and Hedrich, 1992). *Pst* was found to promote IAA production and enable the pathogen to colonize successfully host plants (Chen et al., 2007). In our study, darkness-induced stomatal closure was successfully inhibited by IAA in the wild type but not in *35S::BoTGG1*, indicating that *35S::BoTGG1* was insensitive to IAA-inhibited stomatal closure. Taken together, we speculate that IAA production triggered by *Pst* DC3000 perhaps promotes stomatal reopening and an insensitivity to IAA may contribute to enhanced stomatal defense in *35S::BoTGG1*.

In addition to improving stomatal defense, overexpression of *BoTGG1* should have activated other immune pathway(s), since *35S::BoTGG1* showed significantly higher resistance to *Pst* DC3000 than the wild type in the syringe assay which bypass the stomatal barrier. In *35S::BoTGG1*, aliphatic glucosinolates were significantly reduced due to increased TGG activity, indicating that aliphatic glucosinolate degradation possibly contributes to the enhanced pathogen resistance. Isothiocyanates derived from aliphatic glucosinolates were reported to limit pathogen growth by direct antimicrobial activity for a wide range of bacterial pathogens (Sellam et al., 2007). In addition, TGG mediated degradation of aliphatic glucosinolates is required in programmed cell death during hypersensitive responses upon bacterial inoculation (Andersson et al., 2015). Thus, we speculate that, in addition to stomatal defense, the improved immune response in *35S::BoTGG1* might be due to the increased antimicrobial activity and (or) the activated hypersensitive response.

Overexpressing* BoTGG1* promoted stomatal resistance against bacteria by enhancing the ability of stomata to close. Nevertheless, the stomatal behavior in response to drought stress seemed unaffected, since in *35S::BoTGG1* transpirational water loss from its detached rosette leaves and whole-plant survival under drought stress were similar to wild-type plants. This finding indicates that enhanced stomatal closure behavior may specifically function in the plant defense response against bacterial pathogens, in a way that is distinguishable from other stomatal-related physiological activities responsive to abiotic stress.

Under adverse environmental conditions, plants do not only initiate defense reactions, but also need to coordinate their growth and defense to maximize plant fitness. As a potent defensive compound, glucosinolate and its metabolism play a vital role in biotic stress resistance while also profoundly impacting plant development. Previously, it was found that overexpressing *CYP83B1*, a glucosinolate biosynthesis enzyme that catalyzes indole-3-acetaoxime to indole-3-acetonitrile oxide, causes the early onset of flowering (Naur et al., 2003; Xu et al., 2018). When Jensen et al. (2015) introduced *AOP2* (encoding a 2-oxoglutarate-dependent dioxygenase modifying glucosinolate side chains) into a naturally null Col-0 background, this led to delayed flowering under long-day condition. The double-mutant *myb28/myb29*, which lacks both MYB28 and MYB29, the main regulators of biosynthesis of aliphatic glucosinolates, presents delayed flowering under both short days and long days. Interestingly, under a constant lit condition, the plants with *AOP2* introduced to them were observed to flower earlier, yet *myb28/myb29* showed no alteration in its flowering time (Kerwin et al., 2011). Furthermore, Jensen et al. (2015) showed that the ability of *AOP2* to affect flowering time varies in different accessions due to different genetic backgrounds. Kerwin et al. (2011) study demonstrated that the glucosinolate pathway modulates the plant circadian clock, subsequently leading to complex physiological shifts; however, they also suggested that glucosinolate pathway’s influence on circadian clock does not extend to flowering time. In our study, the expression of *FLC* was significantly higher in late-flowering *TGG1*-overexpressing plants, thus indicating glucosinolate metabolism may regulate flowering time through the *FLC* pathway. In short, the mechanism by which glucosinolates regulate flowering time is quite complex, requiring further study to reveal more about the cross talk between this secondary metabolite and flowering in plants.

**Figure 9:**
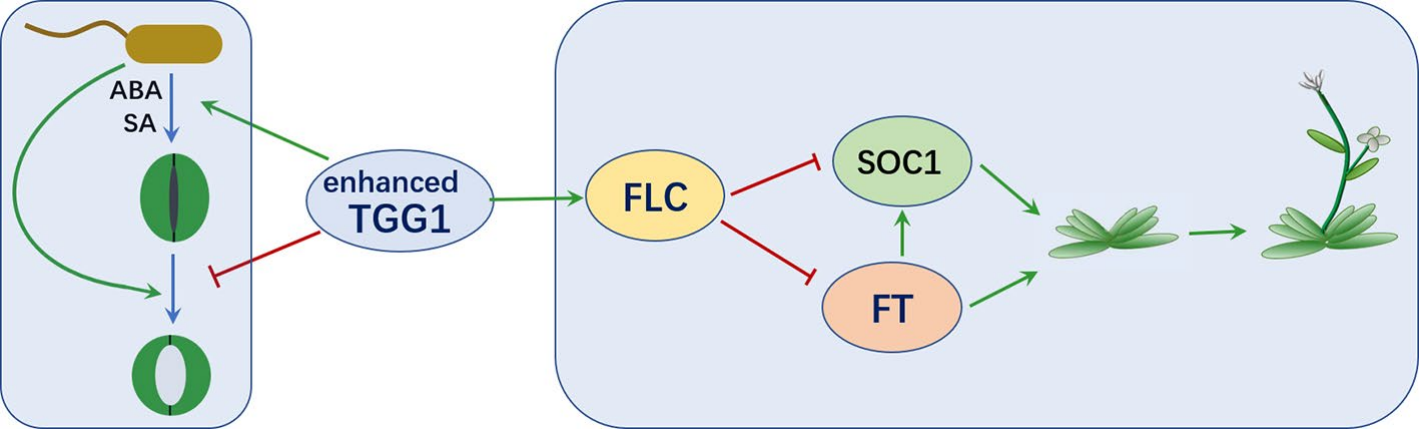
TGG1 modulates stomatal defense and flowering time. Enhanced TGG1 activity improves plant stomatal defense against bacterial pathogens by accelerating stomatal closure and inhibiting the reopening of stomata. TGG1 may modulate flowering time *via* an interaction with the FLC pathway.

In summary, as depicted in **Figure 9**, we have showed that overexpressing a myrosinase gene *TGG1* promoted stomatal defense against a bacterium pathogen in two complementary ways: (1) by accelerating stomatal closure and (2) by inhibiting the reopening of stomata, with former response possibly mediated by ABA and SA. Additionally, the transformation of TGG1 delayed flowering, possibly by promoting the *FLC* pathway.

Due to delayed flowering, the vegetative and total growth phases were prolonged by 9 and 28 days, respectively, which translated into a significant increase in plant biomass. In an applied breeding context, controlling flowering time would be helpful to produce high yields throughout the year. For many Brassicaceae vegetable crops, their late flowering is an important breeding goal; for example, premature bolting is a severe problem in *Brassica rapa* plants—including Chinese cabbage, pak choi, and turnip—as it largely reduces both quality and yield, so extremely late bolting is a major breeding goal in these crops (Kitamoto et al., 2017). Considering that the glucosinolate–myrosinase system is highly conserved between cruciferous crops and the model plant *Arabidopsis*, transformation of *TGG1* might offer a viable and valuable method for breeding late flower varieties to increase their biomass as well as their resistance to bacterial pathogens.

## Data Availability Statement

All datasets generated for this study are included in the manuscript/**Supplementary Files**.

## Author Contributions

JL designed the experiment and KZ conducted the experiment. HS, JZ, WL, and DL participated in various parts of the experiment. JL and KZ wrote the manuscript. All authors have read and approved the final manuscript.

## Funding

This work was supported by the National Natural Science Foundation of China (NSFC) (31570298) and the Heilongjiang Natural Science Foundation (C2017031).

## Conflict of Interest

The authors declare that the research was conducted in the absence of any commercial or financial relationships that could be construed as a potential conflict of interest.

## Supplementary Material

Figure S1 Expression level of BoTGG1 in wild type (WT) and 35S::BoTGG1. Rosette leaves of 4-week-old WT and two independent transgenic lines were harvested for the RT-PCR analysis. ACTIN2 served as the internal control.

Figure S2 Stomatal movement in wild type (WT) and 35S::BoTGG1 upon incubation of water. Leaf peels of WT and 35S::BoTGG1 plants were incubated in water for 3 h and their stomatal apertures were observed and measured every 15 min. (A) The images of representative stomata at each time point during the incubation. (B) Stomatal apertures at each time point during the incubation. Means (± SE) from three independent experiments, each with 100 stomata, are shown.

Figure S3 Phenotypes of wild type (WT) and 35S::BoTGG1 under drought stress. (A) Water loss assay of WT and 35S::BoTGG1 plants. Rosette leaves of 4-week-old WT and 35S::BoTGG1 plants were removed, and detached rosette leaves were weighed at different time points. Means (± SE) from n = 10 individual plants per genotype are shown. (B) Drought resistance of WT and 35S::BoTGG1 plants. Four-week-old WT and 35S::BoTGG1 were treated by withholding water for 2 weeks followed by re-watering.
