# Supplementary figures and images for: Overexpressing the Myrosinase Gene TGG1 Enhances Stomatal Defense Against Pseudomonas syringae and Delays Flowering in Arabidopsis
Source: Front Plant Sci. 2019 Oct 4;10:1230. doi: 10.3389/fpls.2019.01230 (PMC6787276; doi:10.3389/fpls.2019.01230)

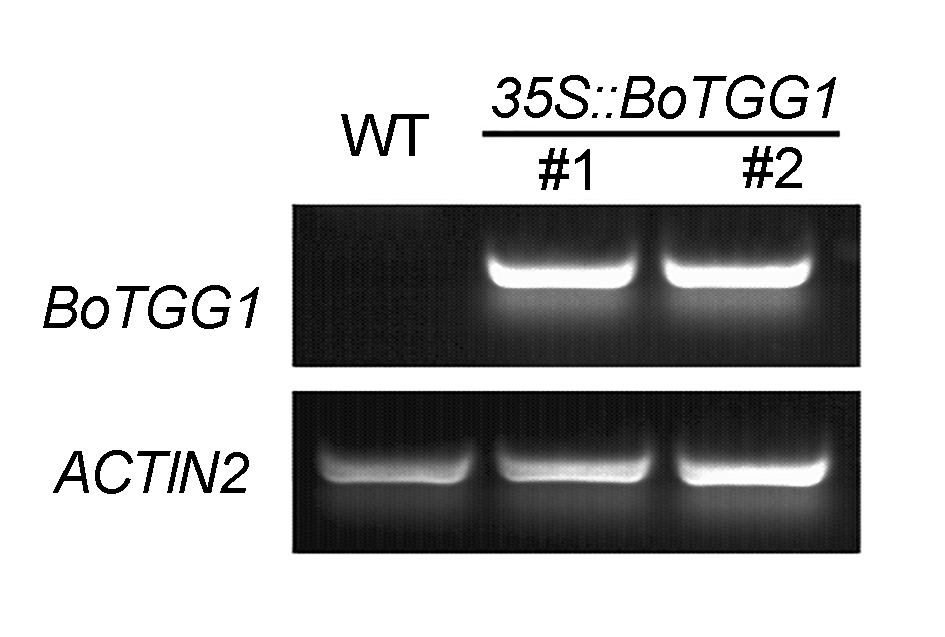

Supplement: Figure S1 — Expression level of BoTGG1 in wild type (WT) and 35S::BoTGG1. Rosette leaves of 4-week-old WT and two independent transgenic lines were harvested for the RT-PCR analysis. ACTIN2 served as the internal control. [file Image_1.tif]

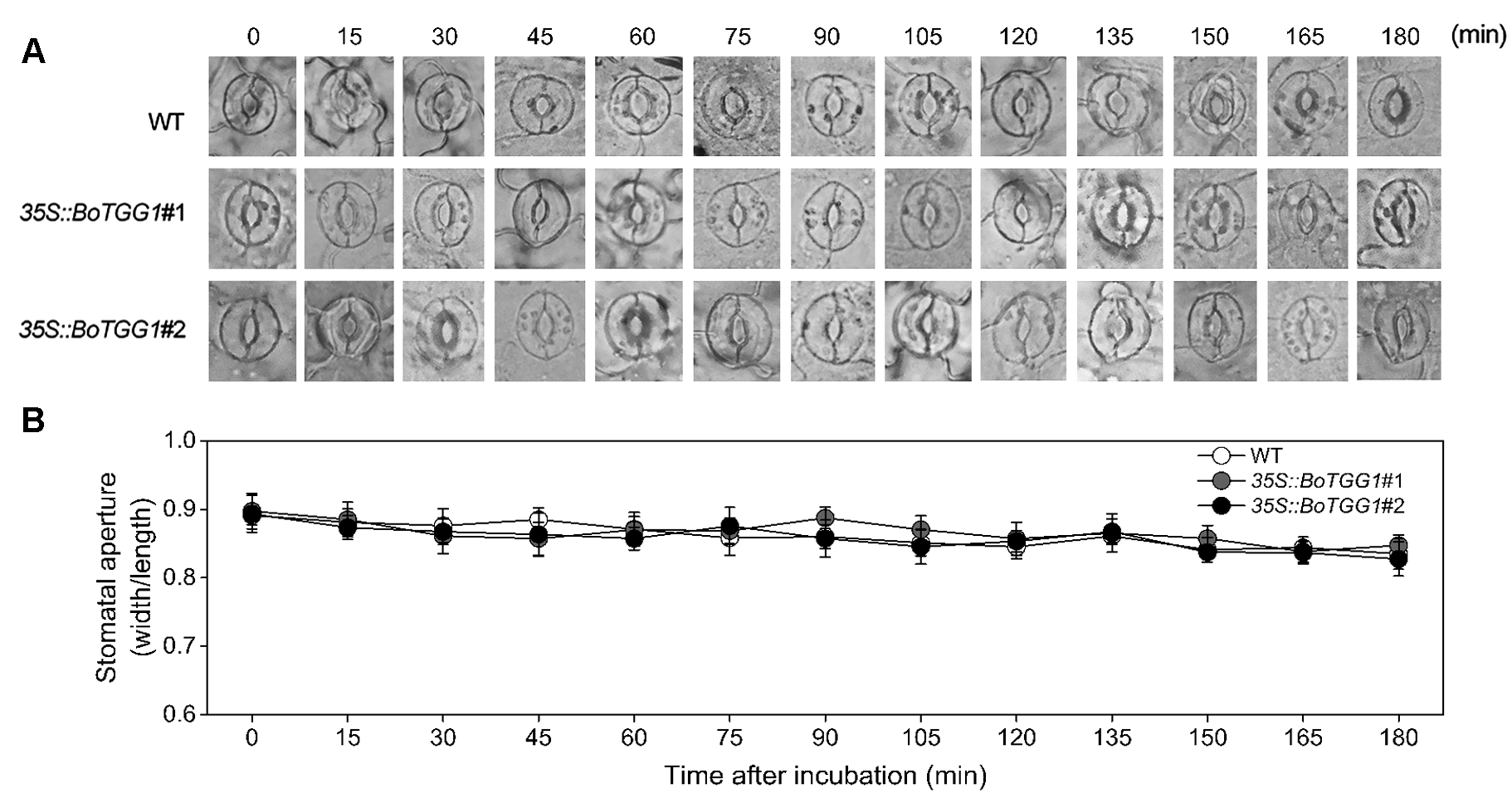

Supplement: Figure S2 — Stomatal movement in wild type (WT) and 35S::BoTGG1 upon incubation of water. Leaf peels of WT and 35S::BoTGG1 plants were incubated in water for 3 h and their stomatal apertures were observed and measured every 15 min. (A) The images of representative stomata at each time point during the incubation. (B) Stomatal apertures at each time point during the incubation. Means (± SE) from three independent experiments, each with 100 stomata, are shown. [file Image_2.tif]

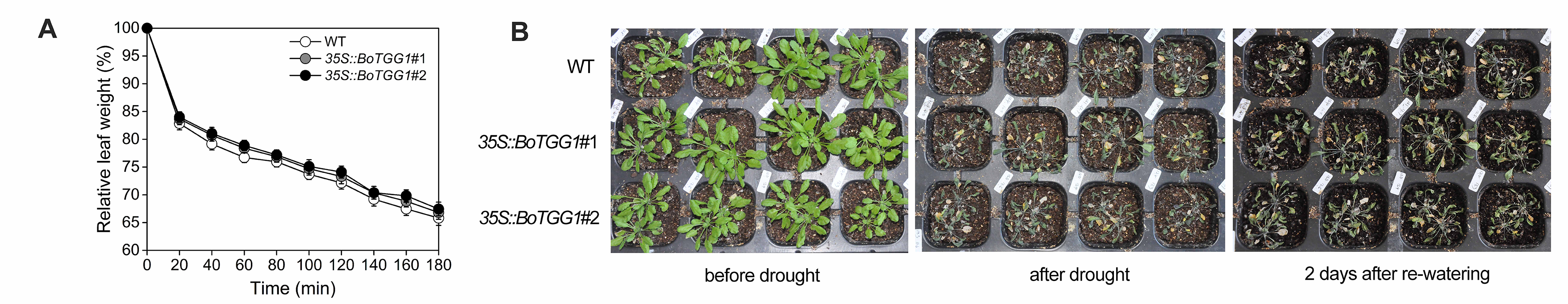

Supplement: Figure S3 — Phenotypes of wild type (WT) and 35S::BoTGG1 under drought stress. (A) Water loss assay of WT and 35S::BoTGG1 plants. Rosette leaves of 4-week-old WT and 35S::BoTGG1 plants were removed, and detached rosette leaves were weighed at different time points. Means (± SE) from n = 10 individual plants per genotype are shown. (B) Drought resistance of WT and 35S::BoTGG1 plants. Four-week-old WT and 35S::BoTGG1 were treated by withholding water for 2 weeks followed by re-watering. [file Image_3.tif]
