# Supplementary material for: Overexpressing the Myrosinase Gene TGG1 Enhances Stomatal Defense Against Pseudomonas syringae and Delays Flowering in Arabidopsis
Source: Front Plant Sci. 2019 Oct 4;10:1230. doi: 10.3389/fpls.2019.01230 (PMC6787276; doi:10.3389/fpls.2019.01230)
Supplement: Supplementary file 4 [file Table_1.docx]

**Table S1 Primer sequences used in the experiment.**

| **Gene name** | **Primer Name** | **Primer Sequence** |
| --- | --- | --- |
| **Primers for gene cloning** | | |
| ***BoTGG1*** | BoTGG1-F | GGCTTAAUATGAAGCTTCTTCATGGACTCG |
|  | BoTGG1-R | GGTTTAAUTCATGCATCAGCGAGCCTCT |
| **Primers for quantitative real-time PCR** | | |
| ***ACTIN2*** | ACTIN2-RT-F | CCATCCTCCGTCTTGACCTT |
| ***FT***  ***FLC***  ***SOC1***  ***MYB28***  ***MYB29***  ***MAM1***  ***CYP79F1***  ***CYP79F2***  ***CYP83A1***  ***FMO_GS-OX1_*** | ACTIN2-RT-R  FT-RT-F  FT-RT-R  FLC-RT-F  FLC-RT-R  SOC1-RT-F  SOC1-RT-R  MYB28-RT-F  MYB28-RT-R  MYB29-RT-F  MYB29-RT-R  MAM1-RT-F  MAM1-RT-R  CYP79F1-RT-F  CYP79F1-RT-R  CYP79F2-RT-F  CYP79F2-RT-R  CYP83A1-RT-F  CYP83A1-RT-R  FMO_GS-OX1_-RT-F  FMO_GS-OX1_-RT-R | ACTTGCCCATCGGGTAATTC  CTAGCAACCCTCACCTCCGA  TCGTAACACACAATCTCATTGCCAAA  TGTGGATAGCAAGCTTGTGG  TAGTCACGGAGAGGGCAGTC  AGCAGCTCAAGCAAAAGGAG  TTGACCAAACTTCGCTTTCA  GCAGATTCGCAATGAAGAGGATAGT GACTTCTTGGGAAACATCGGACATA  TGTGTGGGAGAAGGACTGAAG  CTACAACTCTTTCCACATCGTTT  CCGCAGAAGCTAGAGATTG TCCTCATCCACCTCATTCC  CTTGACGTACTGTCGTTTGTTG GCTACTCCGAATGTTTGATCG  TGATGTGTTTCGACGCTTTG TATAGCGTTTTCGGGCAATG  ATACGGTCCAATCTTGTCATACAGGAT TAATGCCATGTCACGCCTGC  TCTGGAACTACTCATCTAAAGCTGACTCTG ATGTCATGAATGCGTGGCACG |
| ***CYP79B3*** | CYP79B3-RT-F | CTCCTTCTTCCTTGCAAATGGA |
|  | CYP79BE-RT-R | GAGAATCATCAAGAAGCAAAGGG |
| ***CYP83B1*** | CYP83B1-RT-F | GGCAACAAACCATGTCGTATCAAG |
|  | CYP83B1-RT-R | CGTTGACACTCTTCTTCTCTAACCG |
| ***SUR1*** | SUR1-RT-F | CAGGCATATCTAAGGGATGGGTTG |
|  | SUR1-RT-R | AATTATTGTGGCAGGGTCAGGAG |
| ***MYB51*** | MYB51-RT-F | TCAACGAGTTCTTCCTTCGCA |
| ***ABI1***  ***ABI2***  ***PP2CA***  ***LOX1***  ***NPR1***  ***OST1***  ***GHR1***  ***ANAC019***  ***ANAC055***  ***ANACO72*** | MYB51-RT-R  ABI1-RT-F  ABI1-RT-R  ABI2-RT-F  ABI2-RT-R  PP2CA-RT-F  PP2CA-RT-R  LOX1-RT-F  LOX1-RT-R  NPR1-RT-F  NPR1-RT-R  OST1-RT-F  OST1-RT-R  GHR1-RT-F  GHR1-RT-R  ANAC019-RT-F  ANAC019-RT-R  ANAC055-RT-F  ANAC055-RT-R  ANAC072-RT-F  ANAC072-RT-R | ACGGAGGAATCAGAGAACGTG  ATCCTCGCAGCTTCATCTTCTC  TTTCCCGTCTCACATCTTCGTC  CATTCACTGACCCTCACGCC  GAATCTTTCATAGCTCCGTCGC  CGTCGGTTTGTGGTAGAAGA  CCGTCAAAGACACCGTAGAA  TCGCTATGGCAGTTGGCTAAGG  CGATGCGTGTGTTTGCATCCAG  CTTGCGGAGAAGACGACACT  CACCGACGACGATGAGAGAG  ACCAGCAGTGAGTGGTCCAATG  ACCGGAGCCAATATCCTTGACG  TCATGTCAACTACTCTTCACACCCTT  TTCTCCCATTTTGACCATCTTGT  TCTAACCCAAACCGCATCTCGTC  AGTATCCGCAAGACCGTGGAAC  CGTCGAAATGGAAGCACCAAGC  TGTCGACGAACCATTGTTGCTG  ATTATCACGGCGGATGGTCGTC  ACTTGGAGCTTCCATGGCTACG |
